# Effects of Substrate-Binding Site Residues on the Biochemical Properties of a Tau Class Glutathione *S*-Transferase from *Oryza sativa*

**DOI:** 10.3390/genes11010025

**Published:** 2019-12-24

**Authors:** Xue Yang, Jinchi Wei, Zhihai Wu, Jie Gao

**Affiliations:** 1 College of Life Sciences, Jilin Agricultural University, Changchun 130118, China; xueyang840316@163.com; 2 State Key Laboratory of Systematic and Evolutionary Botany, Institute of Botany, the Chinese Academy of Sciences, Beijing 100093, China; 3 College of Biological Sciences and Technology, Beijing Forestry University, Beijing 100083, China; weijc2010@163.com; 4 Faculty of Agronomy, Jilin Agricultural University, Changchun 130118, China; 5 CAS Key Laboratory of Tropical Forest Ecology, Xishuangbanna Tropical Botanical Garden, Chinese Academy of Sciences, Menglun 666303, China; 6 Center of Conservation Biology, Core Botanical Gardens, Chinese Academy of Sciences, Mengla 666303, China

**Keywords:** glutathione *S*-transferase, glutathione-binding site, hydrophobic substrate-binding site, site-directed mutagenesis, enzymatic properties

## Abstract

Glutathione *S*-transferases (GSTs)—an especially plant-specific tau class of GSTs—are key enzymes involved in biotic and abiotic stress responses. To improve the stress resistance of crops via the genetic modification of GSTs, we predicted the amino acids present in the GSH binding site (G-site) and hydrophobic substrate-binding site (H-site) of OsGSTU17, a tau class GST in rice. We then examined the enzyme activity, substrate specificity, enzyme kinetics and thermodynamic stability of the mutant enzymes. Our results showed that the hydrogen bonds between Lys42, Val56, Glu68, and Ser69 of the G-site and glutathione were essential for enzyme activity and thermal stability. The hydrophobic side chains of amino acids of the H-site contributed to enzyme activity toward 4-nitrobenzyl chloride but had an inhibitory effect on enzyme activity toward 1-chloro-2,4-dinitrobenzene and cumene hydroperoxide. Different amino acids of the H-site had different effects on enzyme activity toward a different substrate, 7-chloro-4-nitrobenzo-2-oxa-1,3-diazole. Moreover, Leu112 and Phe162 were found to inhibit the catalytic efficiency of OsGSTU17 to 7-chloro-4-nitrobenzo-2-oxa-1,3-diazole, while Pro16, Leu112, and Trp165 contributed to structural stability. The results of this research enhance the understanding of the relationship between the structure and function of tau class GSTs to improve the abiotic stress resistance of crops.

## 1. Introduction

Glutathione *S*-transferases (GSTs; EC. 2.5.1.18), widely distributed in aerobic organisms, are a superfamily of multifunctional proteins that were found to play an important role in phase II detoxification [1,2,3,4]. During the detoxification process, GSTs conjugate glutathione (GSH) with various electrophilic xenobiotic compounds or endogenous substrates to form soluble nontoxic substances [1]. Plant GSTs also exert other catalytic or non-catalytic functions. As catalysts, GSTs use GSH as a co-substrate or coenzyme to catalyze a variety of reactions including GSH conjugations, GSH-dependent peroxidase reactions, GSH-dependent dehydroascorbate reductase reactions, GSH-dependent isomerizations, and GSH-dependent thioltransferase reactions [1,3,5,6]. Their non-catalytic functions are as binding or carrier proteins for transporting phytochemicals between cellular compartments [1,2,5,7,8,9,10,11,12,13,14]. The various functions are fulfilled selectively during abiotic or biotic stress conditions or normal development. However, only GSH-dependent detoxification received significant attention in plant GSTs to increase the herbicide tolerance of crop in the past decades [3]. In addition to herbicide treatment, other adverse conditions, including oxidative stress, pathogen attack, and an array of abiotic stresses, also cause oxidative injury with an effect on crop yields. Therefore, the focus of research moved on to plant GSTs with GSH-dependent peroxidase (GPOX) activities which are now believed to contribute to defense against oxidative injury during various stresses. GSTs with GPOX activity use GSH as an electron donor to reduce organic hydroperoxides released during oxidative injury to the corresponding monohydroxy alcohols. The plant GSTs with multifunctional GSH-dependent detoxification and GPOX activities have high research value for increasing agricultural plant production. In many studies, enhanced biotic and abiotic stress tolerance in plants was achieved by heterologous overexpression of GSTs [15,16], rather than by enhancing the detoxification or antioxidant capacity of GSTs. Improving the detoxification and antioxidant activity of GSTs by directed mutagenesis is also a method to improve crop tolerance to various stresses [3,17,18,19]. However, successfully modifying GST activity requires understanding the structural basis of GST function.

Based on immunological cross-reactivity, protein sequence similarity, gene structure, substrate specificity, and conservation of specific residues, plant GSTs are subdivided into 14 classes. These include tau (U), phi (F), lambda (L), theta (T), and zeta (Z) GSTs, as well as dehydroascorbate reductases (DHARs), tetrachlorohydroquinone dehalogenases (TCHQDs), g-subunits of the eukaryotic translation elongation factor 1B (EF1Bg), Ure2p, and microsomal prostaglandin E synthases type 2 (mPGES-2). The 14 classes also include recently identified classes such as hemerythrin (H), iota (I) GSTs, and glutathionyl-hydroquinone reductases (GHRs) [20,21,22,23,24,25]. The plant-specific tau class GSTs are the most numerous GST class in plants, and they are essential for plants to resist biotic or abiotic stresses [4]. In addition to its non-catalytic role as a ligand, carrier, or binding protein, tau class GSTs are mainly responsible for GSH-dependent GST detoxification and have GPOX activity to catalyze peroxidation compound reduction [4,26]. Therefore, tau class GSTs are the optional enzymes to be modified for improving their detoxification and antioxidant functions to increase the crop yield. However, the structural basis of tau class GSTs for their detoxification and antioxidant functions is not yet fully understood.

To date, the most accurate three-dimensional structures of tau class GSTs indicate that they are dimerization enzymes about 50 kDa in size. Each subunit consists of two domains with typical structures, including a thioredoxin-folded (βαβαββα) N-terminal domain and an exclusively α-helical C-terminal domain. The domains are connected by a linker loop [27,28]. The conserved N-terminal domain accounts for approximately one-third of the tertiary structure and is responsible for binding deoxidized GSH. The C-terminal domain responsible for binding to electrophilic substrates is more diverse in protein sequence and structure and determines the substrate specificity of enzymes. The active site of each monomer located in a cleft that is formed at the interface of the two domains is composed of amino acids constituting the hydrophilic GSH binding site (the “G-site”) and the hydrophobic substrate-binding site (the “H-site”). Binding GSH and activating sulfhydryl for subsequent nucleophilic attack are the fundamental steps involved in GST catalysis [6,29]. GSH molecules are anchored in the correct position and orientation by electrostatic and hydrogen-bonding interactions determined by conserved amino acids at the G-site. In tau class GSTs, a specific Ser residue at the G-site, which is almost completely conserved, is essential for activating GSH. By deprotonating the thiol, activating GSH carries out nucleophilic attack on the electrophilic center of hydrophobic substrate to detoxicate aryl halides or to act as an electron donor to reduce peroxides [29]. Although the topology and polypeptide sequences involved are quite diverse, the H-site is composed predominantly of hydrophobic residues, which enable a wide range of hydrophobic substrates to identify the H-site accurately in the polar cavity [30]. Compared with the highly conserved G-site, the structural variability at the H-site is more important for the catalytic mechanism.

In a previous study [31], a specific tau class GST (OsGSTU17) was cloned from rice (*Oryza sativa*). This GST showed significant variation in transcription in response to aryl halides, 1-chloro-2,4-dinitrobenzene (CDNB), and hydrogen peroxide treatments. OsGSTU17 protein also showed activities toward aryl halides and peroxides. The predicted structure of OsGSTU17 is similar to that of mango MiGSTU, wheat TaGSTU4, and rice OsGSTU1 (Protein Data Bank (PDB) 5G5F, 1GWC, and 1OYJ, respectively). Based on protein sequence and structure similarity among OsGSTU17, MiGSTU, TaGSTU4, and OsGSTU1, we speculate that Ser15, Lys42, Val56, Glu68, and Ser69 of OsGSTU17 are the G-site residues, while Pro16, Met17, Asn109, Leu112, Tyr113, Phe116, Trp161, Phe162, and Trp165 form the H-site. The role of Ser15 of OsGSTU17 in the activation and stabilization of GSH was demonstrated in the previous study [31]. In this study, we used site-directed mutagenesis to characterize the roles of the other residues of OsGSTU17 at the G- and H-sites on enzymatic activity. This research deepens our understanding of the structural and enzymological properties of the predicted residues involved in GSH and toxic substance binding, and may thereby serve as a basis for improving the abiotic stress resistance of crops.

## 2. Materials and Methods

### 2.1. Protein Sequence Alignments and Structural Modeling of the OsGSTU17 Protein

Protein sequences of representative plant tau class GSTs were selected to align with OsGSTU17 and were further adjusted manually using BioEdit 7.0.5. The structure of OsGSTU17 was built based on the template OsGSTU1 (PDB 1OYJ) crystal structure using InsightII 2005 (Accelrys, Inc., San Diego, CA, USA). The most probable structure of OsGSTU17 was selected according to the model evaluation score calculated by the Profile-3D function of InsightII software.

### 2.2. Site-Directed Mutagenesis of OsGSTU17

To achieve site-directed mutagenesis of OsGSTU17, we performed a two-round polymerase chain reaction (PCR) described by Ho et al. [32]. For the first round of PCR reactions, a pGEM-T/OsGSTU17 plasmid containing the OsGSTU17 complementary DNA (cDNA) was used as the PCR template. Two pairs of primers, each of which included either the reverse mutagenic primer and OsGSTU17-Ex1 or the forward mutagenic primer and OsGSTU17-Ex2 (Table S1, Supplementary Materials), were used to amplify two fragments of OsGSTU17 cDNA with overlapping ends. PCR was performed using Pyrobest^®^ DNA Polymerase (TaKaRa, Dalian, China). Subsequently, purified PCR products were fused to form the completed nucleotide sequences of mutants by recombining them in second-round PCR reactions with primers OsGSTU17-Ex1 and OsGSTU17-Ex2. The purified mutant DNA fragments were digested by *EcoR*I and *Hind*III restriction endonucleases (New England Biolabs Ltd., Beijing, China), and the resulting DNA fragment was recombined into modified pET30a vectors digested with the same restriction enzymes. Modified pET30a vectors (Figure S1) could provide recombined proteins in the correct open reading frame and possess only a six-His tag at the N-terminus, minimizing the effect of N-terminal non-enzymatic amino acids on the correct folding of mutant proteins. The recombinant plasmids were transformed into *Escherichia coli* BL21 (DE3) for prokaryotic expression of the mutant enzymes. The sequences of all mutants were confirmed by sequencing performed by BGI (Beijing Genomics Institute, Shenzhen, China).

### 2.3. Prokaryotic Expression and Purification of the Recombinant Mutant Proteins

The expression, purification, and desalination procedures for all mutant proteins were the same as performed for the wild type [31]. *E. coli* BL21 (DE3) strains containing recombinant expression plasmids were cultured overnight, then diluted 100-fold with Luria-Bertani (LB) medium containing 30 μg∙mL^−1^ kanamycin (Sangon Biotech Co., Ltd., Shanghai, China). Isopropylthio-β-d-galactopyranoside (IPTG), an inducer of protein expression (Sangon Biotech Co., Ltd., Shanghai, China), was then added to the culture medium at a final concentration of 0.1 mM until the bacteria grew to OD_600_ (optical density at 600 nm) = 0.5. After further culturing for 12 h at 37 °C, the bacteria were collected by centrifugation at 5000× *g* for 3 min at 4 °C. The precipitate harvested was resuspended in a binding buffer (20 mM Na_3_PO_4_, 0.5 M NaCl, 20 mM imidazole, pH = 7.4) and disrupted by cold sonication. The homogenate was subjected to centrifugation at 10,000× *g* for 10 min at 4 °C. The resultant particulate material and a small portion of the supernatant were retained for SDS-PAGE analysis to determine whether mutant enzymes were expressed in the soluble form. The remaining supernatant was loaded onto an Ni Sepharose high-performance column (Amersham Pharmacia Biotech, GE Healthcare, Chicago, IL, USA) that was pre-equilibrated with the binding buffer. The mutant proteins with His tags bound to the Ni Sepharose high-performance column were eluted with an elution buffer (20 mM sodium phosphate, 0.5 M NaCl, 500 mM imidazole, pH = 7.4). The purified recombinant proteins were desalted using a PD-10 column (Amersham Pharmacia Biotech) in 10 mM Tris-HCl buffer (pH = 7.4) to reduce the impact of imidazole and salt on the enzymatic reaction. Next, we analyzed the protein samples using electrophoresis with 12% SDS-PAGE to determine purification efficiency. Finally, the concentrations of mutant proteins were measured according to the relative absorbance at 280 nm measured by a Thermo Scientific™ Evolution™ 300 Ultraviolet spectrophotometer.

### 2.4. Substrate Activity

As detoxifying enzymes, tau class GSTs have many substrates. We chose the typical substrates for GST detoxification, including 1-chloro-2,4-dinitrobenzene (CDNB), 7-chloro-4-nitrobenzo-2-oxa-1,3-diazole (NBD-Cl), 4-nitrobenzyl chloride (NBC), 1,2-dichloro-4-nitrobenzene (DCNB), and 4-nitrophenyl acetate (4-NPA) (Sigma-Aldrich Corp., St. Louis, MO, USA). For GOPX detoxification, we chose two substrates, cumene hydroperoxide (Cum-OOH) and ethacrynic acid (ECA) (Sigma-Aldrich), to test the effect of G- and H-site residues on substrate activity. The activities toward CDNB, NBC, DCNB, and ECA were measured as described by Habig et al. [33], the activity toward NBD-Cl was measured as described by Ricci et al. [34], and the activity toward Cum-OOH was measured as described by Edwards and Dixon [35]. The structures of substrates are shown in Table 1. All reactions were carried out at 25 °C using 60 mM GSH (Sangon Biotech). Each reaction was repeated three times, and experimental results were based on the mean values of three independent experiments.

### 2.5. Kinetic Parameters

The kinetic parameters of mutant enzymes were determined using substrates NBD-Cl and GSH. The apparent *K*_m_ and *V*_max_ values for GSH were obtained by measuring the absorbance values at 340 nm for 30 s using GSH concentrations ranging from 0.1 to 1.0 mM and a fixed final NBD-Cl concentration of 1.0 mM. Conversely, the apparent *K*_m_ and *V*_max_ values for NBD-Cl were obtained by measuring the absorbance values at 340 nm for 30 s using NBD-Cl concentrations ranging from 0.1 to 1.0 mM and a fixed final GSH concentration of 1.0 mM. All reactions were carried out at 25 °C. Data were analyzed by non-linear regression analysis implemented by Hyper32 (https://hyper32.software.informer.com/) to obtain the kinetic parameters of mutant enzymes. Each reaction was repeated three times, and experimental results were based on the mean values of three independent experiments.

### 2.6. Thermal Stability

To measure the thermodynamic stability of mutants, thermodynamic stability curves were plotted as a percentage of the catalytic activity toward the CDNB substrate. The catalytic activity of mutants toward the CDNB substrate was tested after a 15-min incubation at a specified temperature from 25 °C to 65 °C with 5 °C intervals. Each reaction was repeated three times, and experimental results were based on the mean values of three independent experiments.

## 3. Results

### 3.1. Identification of Amino Acids Present at the G- and H-Sites of the OsGSTU17 Protein

**Figure 1:**
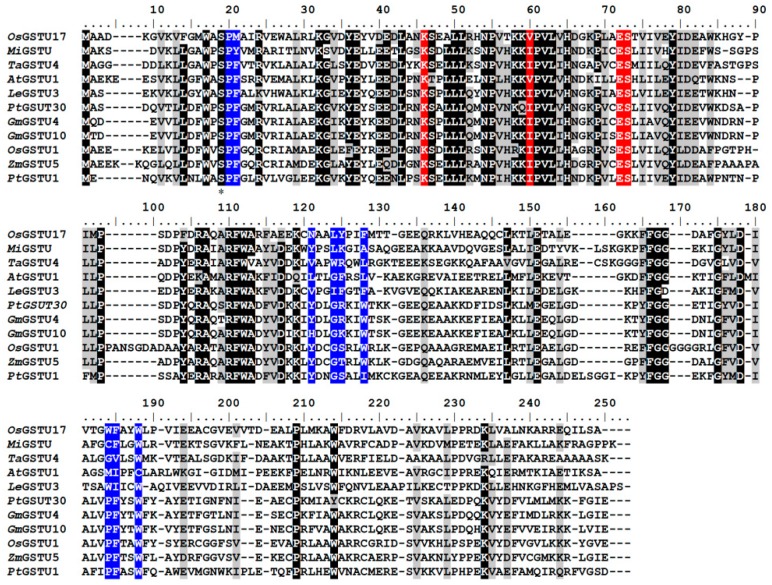
Protein sequence alignment of OsGSTU17 compared with representative plant tau class glutathione *S*-transferases (GSTs). Black squares indicate identity, and gray squares indicate similarity. The active site serine is denoted with *. The G-site amino acids are highlighted by red squares, and the H-site amino acids are highlighted by blue squares. OsGSTU17 (*Oryza sativa*, AF402804), MiGSTU (*Mangifera indica*, 5G5F), TaGSTU4 (*Triticum tauschii*, 1GWC), AtGSTU1 (*Arabidopsis thaliana*, AAL16155), LeGSTU3 (*Lycopersicon esculentum*, AY007560), PtGSTU30 (*Pinus tabulaeformis*, 5J4U), GmGSTU4 (*Glycine max*, 2VO4), GmGSTU10 (*Glycine max*, 4CHS), OsGSTU1 (*Oryza sativa*, 1OYJ), ZmGSTU5 (*Zea mays*, CAA73369), PtGSTU1 (*Pinus tabulaeformis*, AAT69969).

**Figure 2:**
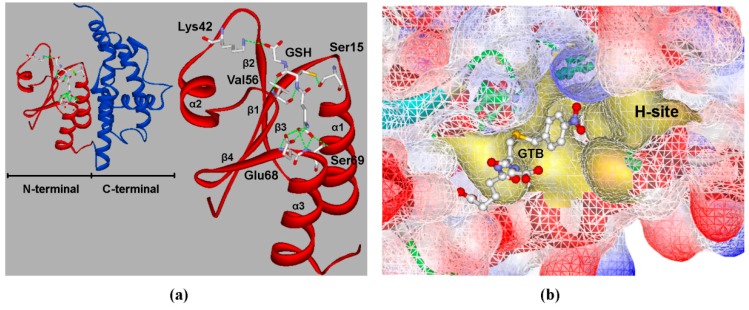
The predicted three-dimensional structure of OsGSTU17 with glutathione (GSH) or *S*-(*p*-nitrobenzyl) glutathione (GTB). Shown are (**a**) the structure of the G-site, and (**b**) the structure of the H-site (indicated in yellow).

A series of tau class GSTs protein sequences were selected for multiple sequence alignment (Figure 1). This alignment showed that Ser19, Lys46, Glu72, and Ser73 were extremely conserved among the conserved N-terminal sequences of tau class GSTs. X-ray structure data of mango MiGSTU (PDB 5G5F), wheat TaGSTU4 (PDB 1GWC), and rice OsGSTU1 (PDB 1OYJ) proteins suggest that very highly conserved Ser19, Lys46, Glu72, and Ser73 residues—as well as a relatively conserved Val60 residue—interact with GSH directly via hydrogen bonding. A three-dimensional structural comparison between OsGSTU17 and the X-ray crystallographic structures revealed that the side-chain groups of conserved amino acids had similar spatial locations and identical extension directions (Figure S2). In a previous study [31], we showed that Ser15 (alignment position 19) of OsGSTU17 is a critical catalytic residue that interacts with the sulfhydryl group of GSH via hydrogen bonding, and the sulfhydryl anion formed provides the nucleophilic attack site of the hydrophobic substrate. We also suggested that other residues that are conserved in OsGSTU17, including Lys42, Val56, Glu68, and Ser69 (alignment positions 46, 60, 72, and 73, respectively) are responsible for enzyme binding with GSH, and function by ensuring that GSH enters the G-site in the correct orientation (Figure 2a).

However, C-terminal sequences are more variable than conserved N-terminal ones (Figure 1), providing the different enzymes with different second-substrate specificities. Hydrophobic xenobiotics interact with the side-chain groups of mainly non-polar amino acids in a pocket structure of the C-terminal domain. The model of OsGSTU17 depicted in Figure 2b shows that Pro16, Met17, Asn109, Leu112, Tyr113, Phe116, Trp161, Phe162, and Trp165 (highlighted in blue in Figure 1) are the amino acids composing the pocket-like H-site. Pro16 and Met17 provide the bottom layer of the pocket, while the sides are comprised of side chains of other residues (Figure S3). Given the importance of key amino acids in these two regions, we mutated Lys42, Val56, Glu68, and Ser69 of the G-site and Pro16, Met17, Asn109, Leu112, Tyr113, Phe116, Trp161, Phe162, and Trp165 of the H-site to Ala through site-directed mutagenesis to investigate the effects of substrate-binding residues on the biochemical properties of OsGSTU17.

### 3.2. Expression and Purification of the Mutant Proteins

To compare the enzymatic properties of mutants and wild-type enzyme, the expression conditions of the mutants were consistent with those of the wild type. The modified pET30a vector (Figure S1) was used as the recombinant prokaryotic expression vector to reduce the effect of unnecessary sequences on protein folding. SDS-PAGE gel electrophoresis (Figure S4) showed that, except for mutant W165A, all mutants of G- and H-sites appeared in the supernatant of *E. coli* BL21 at 37 °C, while the mutant W165A was expressed mainly as an insoluble protein. When the incubation temperature was reduced to 20 °C, the mutant W165A protein was expressed in its soluble form. This finding indicated that Trp165 plays a critical role in the folding of OsGSTU17.

After ultrasonication and centrifugation, we used Ni affinity chromatography to purify the supernatant. Desalination was carried out for recombinant proteins after purification. After desalination, all mutants showed a single band at about 25 kDa in SDS-PAGE (Figure S5) that met the requirements of enzymatic testing.

### 3.3. Substrate Activity of Mutant Proteins

GSTs could catalyze a variety of substrates to bind GSH. Here, we chose two types of substrates (aryl halides and reactive oxygen species, see Table 1) to determine the effects of amino-acid residues on the detoxification and GPOX activity of tau class GSTs. It was shown that wild-type proteins have a strong ability to detoxify 4-chloro-7-nitrobenzo-2-oxa-1,3-diazole chloride (NBD-Cl), 2,4-dinitrochlorobenzene (CDNB), and 4-nitrobenzyl chloride (NBC), and act as an antioxidant in the presence of cumene hydroperoxide. However, wild-type proteins were found to have no activity toward 1,2-dichloro-4-nitrobenzene (DCNB), 4-nitrophenyl acetate (4-NPA), and ethacrynic acid (ECA) [31]. All mutants of the G-site showed a remarkable decline in activity toward two types of substrates, including no activity whatsoever toward cumene hydroperoxide (Table 2). Moreover, K42A mutants only showed slight activity (about 10% of the wild type) toward CDNB, indicating that Lys42 is more important for halogen detoxification among the four amino acids of the G-site. The E68A mutant still retained activity toward three aryl halides, but its activities dropped significantly. V56A and S69A lost activity to NBD-Cl, while they retained 21% and 31% of the activities toward CDNB and 7–8% of the activities toward NBC, respectively, compared with the wild-type enzyme.

The effects of side chains of residues in the H-site on substrate activity are quite different from those of the G-site (Table 3). For the superoxide, cumene hydroperoxide, the activity of all H-site mutants showed an increase of 2.5-fold or more compared to that of the wild type (0.013 μmol∙min^−1^∙mg^−1^), especially the P16A mutant with an approximately six-fold increase. However, all mutants showed sharp decreases in NBC activity and retained a maximum of 20% of that of the wild type. The substitution of each residue of the H-site with alanine enhanced the catalytic activity to CDNB except Trp165. The detoxification activity of P16A, L112A, and W161A mutants to CDNB were obviously elevated compared to wild type. In addition, L112A and W161A mutants showed stronger activity in detoxifying signature substrate NBD-Cl, while the P16A mutant exhibited an approximately five-fold decrease in activity toward NBD-Cl. Moreover, activities of the N109A, Y113A, and F116A mutants toward NBD-Cl were moderately higher than that of the wild-type enzyme. In contrast, we also found slight decreases in the activities of the M17A and F162A mutants toward NBD-Cl. Thus, although the catalytic mechanisms for different halogenated compounds are the same, the effect of mutating a particular residue of H-site varied among different substrates. In addition, the effect of mutating different residues of the H-site on its activity toward a particular substrate also showed significant variation. To conclude, the polar side chains that stabilize GSH binding are more important to the catalytic activity of GSTs than the structure of the H-site, whereas non-polar groups in the hydrophobic pocket determine the specificity toward different substrates.

### 3.4. Kinetic Characterization of the Mutant Proteins

The kinetic constants of mutants were determined using NBD-Cl and GSH as substrates to assess the effects of the different R groups of mutated residues on the kinetic properties of OsGSTU17 (Table 4). A dramatic reduction in activity toward NBD-Cl made it impossible to determine the kinetic parameters of all the G-site mutants, as well as the P16A and W165A mutants of H-site.

Other H-site mutants showed weaker substrate affinity (i.e., higher *K*_m_ values) and lower catalytic efficiency (*k*_cat_/*K*_m_) toward GSH compared to the wild-type enzyme (0.058 mM and 4.552 mM^−1^∙S^−1^). This was especially true for the M17A mutant (0.743 mM and 0.521 mM^−1^∙S^−1^). For NBD-Cl, the affinity and catalytic efficiency of the M17A, N109A, and F162A mutants were markedly reduced. The Y113A and F116A mutants also showed reduced substrate affinity and catalytic efficiency. However, the L112A and W161A mutants showed a unique combination of decreased substrate affinity and increased catalytic efficiency. The substrate affinity of the two mutants was approximately decreased four-fold and their catalytic efficiencies increased from 0.793 mM^−^∙S^−1^ to 1.117 mM^−1^∙S^−1^ and 0.894 mM^−1^∙S^−1^, respectively. This result indicated that the R groups of Leu112 and Trp161 might mainly affect the product release (increased *V*_max_ values). Therefore, when the R groups of these two amino-acid residues were lost, the mutants showed a trend of increasing catalytic efficiency. However, mutants M17A and F162A also showed significantly increased *V*_max_, indicating that these two residues were also involved in the release process of substrate. The catalytic efficiency of mutants M17A and F162A to NBD-Cl decreased due to the influence of R groups on substrate affinity. Meanwhile, the sharply increased turnover number (*k*_cat_) of mutants M17A, L112A, W161A, and F162A implied that the four residues might have a negative influence on the formation of the transition state during catalysis.

### 3.5. Effect of Side-Chain Groups on Thermal Stability of the Enzymes

**Figure 3:**
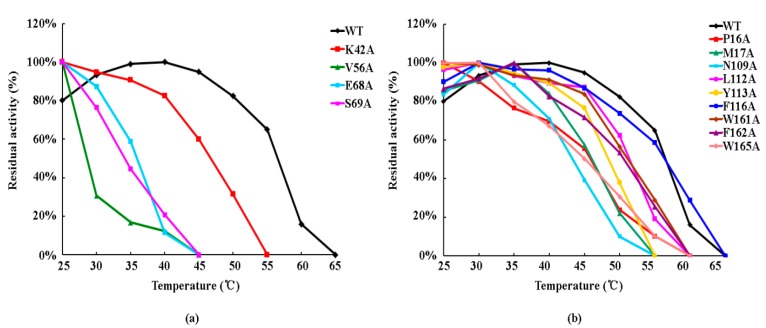
Thermal stability of the wild-type enzyme and OsGSTU17 G- and H-site mutants. (**a**) Thermal stability of G-site mutants; (**b**) thermal stability of H-site mutants. Values of the wild type (detected again) were obtained from Yang et al. [31].

The CDNB and GSH substrates were used to detect the thermal stabilities of mutant enzymes (Figure 3). The maximum activity of the wild-type enzyme was reached at 40 °C, and this point was defined as an activity level of 100%. The wild type was deactivated at 65 °C. The K42A G-site mutant exhibited relatively high stability compared to other G-site mutants and was inactivated at 55 °C, a point at which the wild-type enzyme still retained 65% of the maximum activity. The V56A, E68A, and S69A G-site mutants were highly unstable and lost all activity at 45 °C. At this temperature, the wild-type enzyme retained 95% of its maximum activity. Activity of the V56A mutant showed an especially dramatic drop and retained only 31% of maximum activity at 30 °C. The thermal instability of the G-site mutants suggests that the side-chain groups of amino acids involved in GSH binding are critical for the structural stability of OsGSTU17.

Thermal stability of the F116A H-site mutant was similar to that of the wild type, with both enzymes showing the same inactivation temperature (65 °C) and similar trends (curve shape). Although the deactivation temperatures of the P16A, L112A, W161A, F162A, and W165A mutants were all at 60 °C, the P16A and W165A mutants showed only 20–30% of the maximum activity at 50 °C, while the other mutants had more than 50% of maximum activity. In addition, the M17A, N109A, and Y113A mutants were relatively unstable thermodynamically among all H-site mutants, with an inactivation temperature of 55 °C. This was especially true for the N109A mutant, whose activity sharply declined after 40 °C. These results suggest that the amino acids forming the H-site have less of an effect on the structural stability of OsGSTU17 than the G-site residues.

## 4. Discussion

As phase II detoxification enzymes, glutathione *S*-transferases catalyze the combination of electrophilic toxic compounds with GSH to form hydrophilic compounds. The contaminant halogenated and nitrated compounds are the typical substrates for GSTs which could be used to understand the catalytic mechanism of detoxification of GSTs via a mutation method. As the catalytic mechanism of GPOX activity of GSTs is quite different from that of detoxification, we also chose some peroxides to analyze the catalytic mechanism of GPOX activity of GSTs.

The G- and H-sites which are responsible for GSH binding and hydrophobic substrate binding, respectively, are the structural basis of catalytic activity in each monomer of the dimeric enzyme. Based on structural comparison and sequence alignment, we found that—in addition to Ser15, whose critical catalytic role was demonstrated by a previous study [31]—Lys42, Val56, Glu68, and Ser69 are critical components of the G-site, while Pro16, Met17, Asn109, Leu112, Tyr113, Phe116, Trp161, Phe162, and Trp165 are critical residues of the H-site.

At the G-site, Lys42, Val56, Glu68, and Ser69 all form hydrogen bonds with GSH (Figure 2a), but the hydrogen bonds of different residues have different roles in stabilizing GSH. Lys42 is located between the β2-sheet and α2-helix of OsGSTU17 (Figure 2a). When the side chain of Lys42 was replaced by a methyl group, we observed that mutant K42A almost completely lost activity toward four different substrates (Table 2), probably due to the absence of a hydrogen bond between the ε-amide nitrogen of Lys42 and the carboxyl oxygen of glycine residues present in GSH. We speculate that the hydrogen bond formed by Lys42 is more important for the correct combination and recognition of GSH than other residues of G-site in OsGSTU17. When Val56 was replaced by alanine, we observed a dramatic decrease in the thermodynamic stability (Figure 3a). This phenomenon indicates that the relatively non-conserved Val56 has a significant effect on protein structure. Val56, followed by a highly conserved proline residue (Figure 1), is located just next to the most conserved region of the highly conserved N-terminal domain of all cytosolic GSTs [29]. The most conserved region in OsGSTU17, called the core ββα motif, commences with Pro57 and passes through the β3-sheet (58–62) and β4-sheet (64–66) then finishes up with the end of the α3-helix (69–80). Pro57 having the proper conformation is crucial to the packing of this substructure [27,36]. When the isopropyl group of Val56 was removed, the orientation of Pro57 and the conformation of the core structure underwent great change, but this change did not alter or collapse the important motif responsible for recognition of the γ-glutamyl portion of GSH, permitting some degree of normal binding [29]. Therefore, the activity of mutant V56A toward NBC and CDNB remained at 7% and 21% of the wild type, respectively. In addition, E68A and S69A mutants possessed similar low thermodynamic stabilities. One possible explanation for this is that Glu68 and Ser69 are located in the turn between the β4-sheet and α3-helix, and both interact with the α-amino and α-carboxylate groups of the γ-glutamyl moiety of GSH via hydrogen bonds. Moreover, a conserved electron-sharing network formed by strictly conserved residues Arg18, Glu66, Ser67, and Asp103 of GmGSTU4 could assist the glutamyl γ-carboxylate of GSH to act as a catalytic base accepting the proton from the –SH thiol group of GSH, forming an ionized GSH [26]. Therefore, substitution of either of these adjacent residues with alanine may result in similar distortions of the core motif of OsGSTU17. Finally, we note that all G-site mutants almost completely lost activity toward cumene hydroperoxide, indicating that the effect of GSH on GPOX activity toward peroxidation compounds may be more important than the detoxification of halogenated compounds.

For the H-site, the most critical issue we addressed is how the structure of the H-site determines substrate selectivity. The hydrophobic substrate binding site is shaped like a pocket with bottom and side walls [29]. Side chains of residues which acted as the side walls of the pocket could determine the size of pocket, while amino acids on the bottom of the pocket would affect its depth. To find out which characteristics of the pocket actually affect substrate specificity of OsGSTU17, we examined activity and affinity of H-site mutants toward substrates. According to the predicted structure of OsGSTU17, the R groups of Pro16 and Met17 residues located in the loop linking the β1-sheet and α1-helix comprise the bottom of the H-site (Figure S3). Even though both residues are located in the active loop of OsGSTU17 near the active Ser15 site, they affect substrate activity completely differently. Pro16 is highly conserved in tau class GSTs and is thought to be involved in the catalytic reactions by properly orientating the active loop [23,37]. We speculated that proline may be indirectly involved in GSH activation. Therefore, the substitution of Pro16 with alanine resulted in a 79% decline in the detoxification activity toward NBD-Cl. Interestingly, the P16A mutant protein exhibited different trends in activity toward the typical substrates NBD-Cl and CDNB, showing a three-fold increase in the activity toward CDNB. We compared the crystal structures of *Homo sapiens* GSTM2-2 (PDB 3GUR) with 6-(7-nitro-2,1,3-benzoxadiazol-4-ylthio) hexanol (NBD-Cl analogue, NBDHEX; Figure S6) [38] and *Homo sapiens* hGSTM1a-1a (PDB 1XWK) in the presence of the CDNB analogue 1-glutathionyl-2,4-dinitrobenzene (GS-DNB; Figure S7) [39]. It was found that the spatial position of the electrophilic center of substrate NBD-Cl differed from CDNB when binding with the enzyme, even though they had a similar catalytic mechanism. This might be the reason why the P16A mutant had different trends toward NBD-Cl and CDNB. Sequence alignment results for tau class GSTs (Figure 1) showed that alignment at position 21 generally involved phenylalanine, not methionine. The side-chain group of phenylalanine cannot form a hydrogen bond with Ser69; this suggests that the predicted hydrogen bond formed by Met17 and Ser69 is not necessary to maintain the conformation of the G-site. After mutating Met17, which was also located in the active loop, we observed a slight decrease in activity toward NBD-Cl (Figure 2). Although the formation of transition state and the release of product were improved, the change in OsGSUT17 protein structure caused by methionine substitution seriously affected the affinity of mutant M17A toward GSH and NBD-Cl, which resulted in a significant reduction in the catalytic efficiency of mutant M17A (Table 4). As with Pro16, Met17 was speculated to hinder the binding of GSH and CDNB, resulting in increased activity of M17A mutant toward CDNB.

The residues constituting the side walls of the pocket could be divided into three categories based on the activity of mutants toward NBD-Cl and CDNB. In the first category, the substitution of Trp165 sharply lowered the activity of OsGSTU17 toward NBD-Cl and CDNB. The structure of OsGSTU17 indicated that the distance between Trp165 and Ala14 located in the N-terminal domain was short enough for the formation of hydrogen bonds, which could stabilize the relative positions of the N-terminal and C-terminal domains. This structural change not only affected the activity of enzyme to substrates NBD-Cl and CDNB, but also led to the poor stability of protein structure. Therefore, the phenomenon that the mutant W165A was only expressed in soluble form at 20 °C was observed.

In the second category, the deletion of side-chain groups of residues Leu112 and Trp161 significantly elevated the activity of the enzyme toward the two substrates (Table 3) and improved the catalytic efficiency toward NBD-Cl. We speculated that the pocket side wall composed by Leu112 and Trp161 blocked the formation of transition state and the release of product. Therefore, when the side-chain groups were removed, the mutants showed increased substrate activity, *V*_max_, and *K*_cat_ (Table 3 and Table 4). Meanwhile, we found that Leu112 and Ile115, both located in the α4-helix, had hydrophobic interactions with Ile169 and Leu166, both located in the α7-helix. In the hydrophobic collapse model, hydrophobic interactions are considered to have crucial effects on protein folding [40]. The substitution of Leu112 destroyed the structure of the hydrophobic zipper, making the mutant protein more prone to precipitation when expressed at 37 °C. Therefore, properties of the enzyme were analyzed by using the mutant protein expressed at 20 °C. A similar hydrophobic core formed by Leu located in α5 and another conserved Leu in the C-terminal domain was also found in pi, mu, and α classes of GSTs [27].

Finally, Asn109, Tyr113, Phe116, and Phe162 belong to the third category. The substitution of alanine for these residues did not strongly affect enzyme activity toward NBD-Cl or CDNB. A possible contribution of the side-chain group of Tyr113 was to inhibit egress of the products from the active site [41]. However, the *V*_max_ of mutant Y113A had no obvious decreasing trend. The Phe162 of OsGSTU17 had little effect on substrate activity, but had a significant effect on substrate binding, product release, and transition state transformation, especially for substrate NBD-Cl binding (approximately 10-fold change in *K*_m_). The Asn109 and Phe116 in OsGSTU17 had similar effects on the catalytic mechanism (similar substrate activity and kinetic parameters), but they were quite different in the role of thermodynamic stability. Asn109 was more conducive to protein stability. In addition to the residues of the H-site predicted above, there may be other amino acids involved in the interaction between enzyme and hydrophobic substrate. For example, Lo et al. [42] found that two tau GSTs with only three amino acids (position 89, 117, and 172) difference in protein sequences in sweet orange showed great difference in catalytic efficiency toward CDNB, while the three residues located in the C-terminal domain were far away from the generally accepted H-site. Site-directed mutagenesis was used to address questions regarding the functional roles of these H-site residues. Glu117 had a critical contribution to the recognition of CDNB in GSTU1, while Arg89 in GSTU1 might maintain the architecture of the G-site. Interestingly, the mutant enzyme RKV was able to conjugate 4-NPB to GSH, whereas the wild type was not. This indicated that Val172 had a crucial role in creating the active site architecture that was suitable for accommodating other substates [42]. However, in our research, the residue with a similar function to Val172 was not found in OsGSTU17. Therefore, it is necessary to further explore the amino acids of GSTs responsible for hydrophobic substrates and analyze their roles in enzymatic reactions.

NBC is a small-sized molecular substrate of GSTs. The substitution of residues of the H-site would enlarge the pocket, resulting in deleterious structural changes of OsGSTU17 with respect to NBC, such as a failure to anchor the NBC substrate in the proper orientation and affecting nucleophilic attack with GSH. Thus, the activity of all the mutants of H-site toward NBC decreased dramatically (Table 3).

As a model substrate, cumene hydroperoxide is used extensively to detect the GPOX activity of GSTs [43]. The catalytic mechanism of GSTs toward cumene hydroperoxide is different from detoxification. GSTs reduce cell damage by reducing cumene hydroperoxide to the corresponding non-toxic alcohol cumenol. By contrast, cumene hydroperoxide is not a favored substrate of OsGSTU17. However, all the mutants of the H-site showed higher activity toward cumene hydroperoxide. In particular, the P16A, L112A, and F162A mutants showed 6.2-fold, 5.1-fold, and 4.1-fold higher activity toward cumene hydroperoxide than the native enzyme, respectively. In view of the large space occupied by cumene hydroperoxide, we speculated that the residues of the H-site hindered the entry of cumene hydroperoxide into the H-site. Strikingly, a replacement of Met232, which the side chain pointing away from the pocket, by Ala in mice GSTT1-1 led to an approximately 30-fold increase in catalytic activity toward cumene hydroperoxide [44]. This result implies that residues affecting the substrate activity of cumene hydroperoxide are not necessarily those composing the H-site. Any amino acids that affect the shape of the pocket may be the candidate residues for regulating the substrate specificity of OsGSTU17.

The results of this article provide significant new data and improve our understanding of the structure and function of tau class GSTs. With the exception of catalytic residue Ser15, G-site residues influence enzymatic properties by stabilizing GSH though hydrogen bonds or by maintaining protein configuration. H-site residues determine substrate activity, catalytic efficiency, and substrate specificity by regulating the shape of the pocket. The influence of residues at the H-site on enzyme characteristics is more complex than those at the G-site, which needs further study. Using this knowledge, the artificial directional modification of the enzymatic properties of GSTs could probably be achieved using site-directed mutagenesis. Combined with molecular breeding technologies, the artificial directional modification of GSTs may provide an alternative direction for GST application in agricultural improvement or environmental protection [25,28,45,46].

## 5. Conclusions

In this study, we predicted the glutathione and hydrophobic substrate-binding sites of OsGSTU17, and successfully modified amino acids present in these two sites by site-directed mutagenesis. Mutants proteins were expressed in a prokaryotic system, and their enzymatic properties were examined after purification. These properties were found to clearly explain the effect of the side-chain groups of residues on the catalysis and structure of OsGSTU17. Lys42, Val56, Glu68, and Ser69, which were predicted to be involved in the G-site, were found to be responsible for interacting with GSH via hydrogen bonding. Significant changes in substrate activity indicated that the hydrogen bonds formed between Lys42 and GSH were essential for the correct orientation of GSH. In addition, Val56, Glu68, and Ser69 were found to have great influence on the thermal stability of the protein structure. Among the H-site residues, Trp165 appeared to control the connection between the G- and H-sites and maintain the spatial conformation of the active site. With respect to aryl halide substrates, OsGSTU17 required a link between the G- and H-sites to complete the detoxification reaction. However, the enlargement of the active cavity was more conducive to the reduction of peroxides. With respect to the detoxification activity of GSTs, different halogenated aromatic compounds have different conformational requirements for the H-site. Pro16, Leu112, and Trp161 could be considered as the residues to be modified to enhance the substrate activity and catalytic efficiency of OsGSTU17. In conclusion, our results partially reveal the relationship between the structure and function of tau class GSTs.

## Figures and Tables

**Table 1:** The structures of substrates of OsGSTU17 mutants.

NBD-Cl	CDNB	NBC	Cumene Hydroperoxide
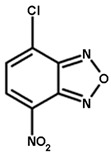	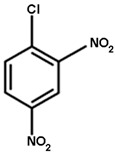	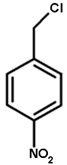	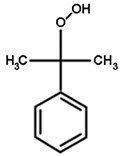

Note: The structures of substrates that showed no activity are not shown. NBD-Cl is short for 7-chloro-4-nitrobenzo-2-oxa-1,3-diazole; CDNB is short for 1-chloro-2,4-dinitrobenzene; NBC is short for 4-nitrobenzyl chloride.

**Table 2:** Specific activities of OsGSTU17 G-site mutants.

Substrate	Specific Activity (μmol min^−1^ mg^−1^)					
	WT [31]	S15A [31]	K42A	V56A	E68A	S69A
NBD-Cl (10 mM)	0.203 ± 0.006	ND	<0.01	<0.01	0.016 ± 0.002	<0.01
CDNB (60 mM)	0.113 ± 0.019	ND	0.011 ± 0.001	0.024 ± 0.002	0.029 ± 0.002	0.035 ± 0.002
NBC (60 mM)	1.153 ± 0.046	ND	ND	0.082 ± 0.014	0.063 ± 0.010	0.101 ± 0.016
Cum-OOH (69 mM)	0.013 ± 0.002	ND	<0.01	ND	<0.01	ND

Note: Values shown are means ± SD, as calculated from three independent replicates. ND indicates that no activity was detected. Values of wild-type (WT) and mutant S15A proteins (detected again) were obtained from Yang et al. [31].

**Table 3:** Specific activities of OsGSTU17 H-site mutants.

Substrate	Specific Activity (μmol∙min^−1^∙mg^−1^)									
	WT [31]	P16A	M17A	N109A	L112A	Y113A	F116A	W161A	F162A	W165A
NBD-Cl (10 mM)	0.203 ± 0.006	0.041 ± 0.002	0.183 ± 0.001	0.209 ± 0.002	0.380 ± 0.015	0.224 ± 0.005	0.246 ± 0.005	0.267 ± 0.003	0.192 ± 0.001	0.049 ± 0.009
CDNB (60 mM)	0.113 ± 0.019	0.349 ± 0.008	0.159 ± 0.003	0.189 ± 0.014	0.245 ± 0.001	0.129 ± 0.005	0.138 ± 0.001	0.347 ± 0.001	0.129 ± 0.001	0.080 ± 0.003
NBC (60 mM)	1.153 ± 0.046	0.017 ± 0.015	0.081 ± 0.012	0.083 ± 0.004	0.237± 0.040	0.069 ± 0.024	0.088 ± 0.003	0.164 ± 0.092	0.081 ± 0.001	0.189 ± 0.049
Cum-OOH (69 mM)	0.013 ± 0.002	0.081 ± 0.008	0.034 ± 0.011	0.032 ± 0.002	0.066 ± 0.004	0.032 ± 0.001	0.033 ± 0.003	0.037 ± 0.002	0.053 ± 0.002	0.028 ± 0.003

Note: Values shown are means ± SD, as calculated from three independent replicates. Wild-type values (detected again) were obtained from Yang et al. [31].

**Table 4:** Kinetic analysis of the OsGSTU17 H-site mutants.

	GSH				NBD-Cl			
	*K*_m_	*V*_max_	*k*_cat_	*k*_cat_/*K*_m_	*K*_m_	*V*_max_	*k*_cat_	*k*_cat_/*K*_m_
	(mM)	(μM∙min^−1^∙mg^−1^)	(S^−1^)	(mM^−1^∙S^−1^)	(mM)	(μM∙min^−1^∙mg^−1^)	(S^−1^)	(mM^−1^∙S^−1^)
WT [31]	0.058 ± 0.006	0.225 ± 0.011	0.264	4.552	0.324 ± 0.016	0.219 ± 0.006	0.257	0.793
M17A	0.743 ± 0.049	0.397 ± 0.018	0.387	0.521	3.661 ± 0.843	0.830 ± 0.168	0.809	0.221
N109A	0.192 ± 0.003	0.268 ± 0.003	0.196	1.019	0.781 ± 0.034	0.364 ± 0.008	0.266	0.341
L112A	0.307 ± 0.010	0.485 ± 0.001	0.946	1.579	1.487 ± 0.079	0.896 ± 0.019	1.750	1.177
Y113A	0.120 ± 0.004	0.260 ± 0.003	0.220	1.842	0.471 ± 0.012	0.327 ± 0.008	0.277	0.589
F116A	0.166 ± 0.007	0.286 ± 0.005	0.322	1.942	0.882 ± 0.028	0.455 ± 0.014	0.512	0.581
W161A	0.221 ± 0.005	0.341 ± 0.006	0.573	2.588	1.117 ± 0.022	0.594 ± 0.011	0.999	0.894
F162A	0.360 ± 0.013	0.233 ± 0.003	0.421	1.170	3.866 ± 0.871	0.869 ± 0.198	1.573	0.407

Note: Values shown are means ± SD, as calculated from three independent replicates. The values of the wild-type enzymes (detected again) were obtained from Yang et al. [31].

## Supplementary Materials

The following are available online at https://www.mdpi.com/2073-4425/11/1/25/s1, Figure S1: The cloning/expression region of modified pET30a vector for prokaryotic expression of mutants; Figure S2: The representation of overlap of protein structures among OsGSTU17, MiGSTU, TaGSTU4, GmGSTU4, and OsGSTU1 with inhibitor GSX; Figure S3: The representation of the predicted model of the H-site of OsGSTU17 with inhibitor Nb-GSH according to the crystal structure of GmGSTU4 (PDB 2VO4); Figure S4: SDS-PAGE analysis for the expression of recombinant mutant proteins; Figure S5: SDS-PAGE analysis for the purification of recombinant mutant proteins; Figure S6: The representation of the crystal structure of *Homo sapiens* GSTM2-2 with NBD-Cl analogue (NBDHEX); Figure S7: The representation of the crystal structure of *Homo sapiens* hGSTM1a-1a with CDNB analogue (GS-DNB); Table S1: Primers for OsGSTU17 mutants.
